# Treatment of Liver Metastases From Colorectal Cancer: Medico-Surgical Strategies

**DOI:** 10.4021/gr317e

**Published:** 2011-05-20

**Authors:** Ismail Essadi, Yassir Sbitti, Mohamed Fetohi, Khaoula Alaoui Slimani, Meryam Essadi, Elmehdi Tazi, Mohamed Ichou, Hassan Errihani

**Affiliations:** a Department of Medical Oncology at the Military Hospital Mohamed V, Rabat, Morocco; b National Institute of Statistics and Applied Economics (INSEA), Rabat, Morocco; c Department of Medical Oncology at the National Institute of Oncology, Rabat, Morocco

**Keywords:** Metastasis, Liver, Surgery, Chemotherapy, Radiofrequency, Colorectal cancer

## Abstract

**Background:**

The management of hepatic metastases from colorectal cancer can be understood only as part of a multidisciplinary strategy. Progress experienced by medical treatment, surgical techniques and ways of imaging, has improved the prognosis of patients with liver metastases of colorectal cancers. This work displays the experience of Medical Oncology unit at the Military training hospital in Rabat.

**Methods:**

From January 2007 to December 2009, 60 patients with liver metastases from colorectal cancer, synchronous or metachronous were supported in the Medical Oncology unit at the Military training hospital in Rabat.

**Results:**

Liver metastases were synchronous in 41 (68%) patients and metachronous in 19 (32%). Patients were classified into 3 categories according to their resectability: 14 (22%) were resectable at the outset, 28 (47%) were unresectable and 18 (31%) were considered uncertain resectability. Thirty-five patients (58%) received neoadjuvant chemotherapy before surgical gesture, 25 (42%) received chemotherapy after resection of primary tumor. This chemotherapy enabled the resection of liver metastases in 5 patients initially deemed uncertain resectability. The average objective responses to chemotherapy were in the range of 59% with 4 complete responses and one confirmed histologically. Twenty-three patients (38%) underwent surgery including 15 liver resections with R0 (25%). The median progression-free survival in this series was 15.5 months. Some minor side effects were noted, which have not entered the prognosis of patients.

**Conclusions:**

Hepatic resection remains the only potentially curative treatment of liver metastases of colorectal cancers. Perioperative chemotherapy is a promising standard, which has improved the prognosis of patients historically associated with a poor prognosis.

## Introduction

Colorectal cancer is the 4th in order of frequency of cancer worldwide [1]. The Liver is the most common site of metastases, since all venous confluences from the gastrointestinal tract converge on this gland [1].

Surgery of liver metastases of colorectal cancer (LMCRC) undeniably improved patient survival, as well as the use of targeted therapies, which has revolutionized the management of LMCRC [2], which can not be conceived outside a multidisciplinary approach at large term.

## Materials and Methods

This is a retrospective study, conducted over a period of three years from January 2007 to December 2009.

Inclusion criteria: any patient with cancer of the colon or rectum, and associated with histologically confirmed liver metastases, whether synchronous or metachronous. The patient’s general condition must be maintained with a performance status score of 0, 1 or 2.

Exclusion criteria: any patient with poor general condition.

## Results

Sixty patients were treated for LMCRC between January 2007 and December 2009, including 34 men and 26 women, with a sex ratio 5M/4W.

The average age was 49.5 years. The primary tumor was colon in 35 patients (58%) and rectal in 25 (42%). Identification of K-RAS status was performed in nineteen patients. The results were in favor of K-RAS mutations in 16 patients, the other 3 were K-RAS wild. Twenty-five patients (42%) have undergone an initial surgery on the primary tumor, including thirteen (22%) in emergency under the care of an occlusive syndrome. The Liver metastases (LM) were synchronous in 41 patients (68%) and metachronous in 19 (32%). These (LM) were considered resectable immediately in 14 patients (22%), unresectable in 28 patients (47%), and uncertain resectability in 18 patients (31%). Thirty-five patients (58%) received neoadjuvant chemotherapy before surgery. Twenty-five patients (42%) received chemotherapy after resection of primary tumor.

All our patients received chemotherapy with bevacizumab, in combination with fluoropyrimidine intravenously or orally, plus Irinotecan or Oxaliplatin, with cross-over in case of tumor progression. Average objective response was 59%, with 4 complete responses in liver metastases, and one of them was confirmed histologically. Tumor stabilization was observed in 21% of patients, and progression in 34%. The median duration of progression-free survival was 15.5 months. Twenty-three patients received 38% liver surgery. Resection R0 was found in 15 patients (25%) and R1 in eight patients (13%).

One patient underwent a radiofrequency on liver damage with good evolution.

No patient judged operable immediately progressed under chemotherapy.

Five patients (8%) considered unclearly resectable, became operable after chemotherapy. No patient initially considered inoperable could be made on liver metastases after chemotherapy. The rate of carcinoembryonic antigen (CEA) was elevated in twenty-five patients (41%). Normalization of these levels was noted in seventeen patients (28%) after chemotherapy. Some side effects of chemotherapy were identified. Intestinal perforation was noted in one patient (1.7%), it has been assumed in the emergency operating room, with good evolution. A single case of gastrointestinal bleeding had such great abundance of rectal bleeding that led to a blood transfusion without commitment prognosis. Two cases of venous thrombosis (3.5%), presumably due to bevacizumab, have been successfully treated with low molecular weight heparin, and one case of massive proteinuria without impact on renal function. In all these cases, discontinuation of Bevacizumab has been decided. Hand-foot syndrome grade 2 was detected in three patients (5%) and diarrhea grade 2 in 19 patients (32%) were fully managed by medical treatment, and had resulted in no change in dose. Cons by persistent grade 2 neuropathy with functional impairment were registered in seven patients (11.5%) leading to discontinuation of oxaliplatin.

## Discussion

Hepatic metastases occur in 25% of patients with colorectal cancers diagnosed advanced stage [1]. There are synchronous in 20% of cases and metachronous in 75% of cases [1]. In this series, the figures are the opposite of what is described in the literature, probably because the diagnosis is often delayed. Resection of liver metastases completely changes the prognosis of the disease, making it curable [2]. The management of patients experienced in recent years considerable progress on several levels, tripling the length of patient survival [3]. First, the development of the concept of targeted therapies with novel molecules, based on sound biological solids (Anti-Angiogenic, anti-EGFR); then the establishment of new therapeutic strategies, taking into account the peculiarities of the disease and the toxicity of treatment (therapy and chemotherapy to break the card); and finally changing members involving the patient as a partner in his own care [4]. The generalization of multi-disciplinary consultation meetings and the establishment of the Cancer Plan by the political authorities have defined a roadmap for the multidisciplinary care of Liver metastasis. R0 resection of LM, offers a great chance of cure [5]. This resection should be discussed on the technical and oncological criteria [6]. The value of perioperative chemotherapy is to enhance the chances of resectability of LM [7]. A phase III study of the EORTC, which enrolled 364 patients, compared two arms of LM surgery alone and three months of chemotherapy according to the FOLFOX-4 before and after surgery of LM. The results were in favor of an absolute benefit of 9.2% in terms of progression-free survival at 3 years for the arm containing the peri-operative chemotherapy [8]. Indeed, it allows the eradication of micro-metastatic disease, chemotherapy sensitivity testing of LM and to predict their evolutionary potential [9]. That said, the choice of treatment and treatment sequence is a real dilemma. It should optimize the patient’s survival without neglecting his comfort, taking into account the oncologic status, the patient’s priorities, and feasibility criteria of treatment [10]. In this series, all patients who received liver resection underwent a peri-operative chemotherapy, which allowed the conversion of nine patients initially deemed unclear operability, thus benefiting from curative resection. A more aggressive approach would also convert 15% of LM deemed inoperable at the beginning, operable lesions, at the cost of greater toxicity [11, 12]. Side effects of chemotherapy in this series were under control.

### Conclusion

This is a young but promising experiment, with results concordant with the literature. Liver metastases surgery remains the cornerstone of treatment. Peri-operative chemotherapy has become a standard treatment. Molecular biology represents a kind of radar for targeted therapies, to detect molecular abnormalities. The future will bring the results of tests that will define the strategic real impact of each treatment sequence.
